# Environmental enrichment enhances anesthetic actions in rat amygdala hippocampal circuits *in vitro*

**DOI:** 10.3389/fphar.2025.1732630

**Published:** 2025-12-19

**Authors:** Kenta Onishi, Rika Sasaki, Koki Hirota, Tomonori Takazawa

**Affiliations:** Department of Anesthesiology, Faculty of Medicine, University of Toyama, Toyama, Japan

**Keywords:** desflurane, environmental enrichment, GABAergic inhibition, general anesthetics, hippocampus, propofol

## Abstract

**Introduction:**

Environmental enrichment enhances hippocampal synaptic plasticity, yet its influence on anesthetic action remains poorly understood. This study tested the hypothesis that enriched environment (EE) rearing modifies the inhibitory effects of propofol and desflurane on synaptic transmission within a novel limbic circuit slice preparation preserving amygdala–hippocampal connections.

**Methods:**

Slices were obtained from male rats reared in either a standard environment (SE) or an EE. Electrophysiological recordings measured population spike (PS) amplitudes in CA1 pyramidal neurons.

**Results:**

In slices from EE rats, the inhibitory effects of both anesthetics on PS amplitude were markedly potentiated compared with SE rats. The IC50 of propofol decreased from 3.3 × 10–4 M [IQR: 2.7 × 10–4 −3.5 × 10–4] in SE to 5.4 × 10–5 M [IQR: 5.1 × 10–5 −5.6 × 10–5] in EE (P = 0.002), whereas that of desflurane decreased from 10.4 vol% [IQR: 10.3−11.5] to 6.5 vol% [IQR: 4.8−7.1] (P = 0.002). Potentiation was more pronounced for propofol, which acts primarily through GABA receptors, whereas desflurane, with multiple molecular targets, showed a smaller potency change accompanied by an increased Hill coefficient, suggesting altered receptor binding cooperativity. Recovery of inhibitory tone following stimulus-induced disinhibition was accelerated in EE slices. This effect was most prominent with propofol, for which the recovery time constant decreased from 146.0 s [IQR: 110.6−642.8] in SE to 36.6 s [IQR: 25.5−48.9] in EE (P = 0.008).

**Discussion:**

These findings demonstrate that rearing in an enriched environment enhances anesthetic potency by strengthening GABAergic inhibitory circuits and modifying pharmacological profiles at the network level. This enhancement was most evident for propofol, indicating that environmental factors can significantly influence anesthetic sensitivity within hippocampal circuits. Clinically, an individual’s life history and environment may represent critical yet overlooked determinants of anesthetic requirements. These results highlight the importance of personalized pharmacology in anesthesia and suggest that standardized dosing of GABAergic agents may cause overdose in individuals with enhanced inhibitory function. Overall, this study provides mechanistic insights into how environmental neuroplasticity modulates anesthetic pharmacodynamics, advancing our understanding of interindividual variability in drug response and perioperative safety.

## 1 Introduction

The variability in individual responsiveness to general anesthetics remains a critical issue in clinical practice. While pharmacokinetic factors often explain differences in dosing, growing evidence suggests that an individual’s environmental background and lifestyle factors significantly modulate neural sensitivity to anesthetics (pharmacodynamics) (Kil et al., 2012). Recently, environmental enrichment, a paradigm providing sensory, cognitive, and motor stimulation, has gained attention for its impact on the central nervous system and its potential interaction with anesthetic outcomes (Van Praag et al., 2000; Zhao et al., 2015). A comprehensive review highlighted that environmental enrichment exerts neuroprotective effects and modulates cognitive recovery following anesthesia, suggesting a profound interaction between environmental factors and anesthetic mechanisms (Chang and Tian, 2022). Furthermore, clinical studies have indicated that socioeconomic and educational backgrounds, factors reflecting aspects of human environmental enrichment, significantly influence patients’ preoperative anxiety and concerns regarding anesthesia (Al Modanat et al., 2024). This suggests a translational link whereby environmental history modulates internal emotional state relevant to anesthetic management. A key physiological effect of environmental enrichment is its robust anxiolytic and stress-reducing property. Previous behavioral and neurochemical studies have demonstrated that animals reared in an enriched environment (EE) exhibit reduced anxiety-like behaviors and altered stress responses compared with those in standard environments (Fox et al., 2006; Simpson and Kelly, 2011). These behavioral changes are often accompanied by functional modifications in the limbic system, particularly within the amygdala and hippocampus (Eckert et al., 2010; Ashokan et al., 2016). The hippocampus is essential for memory consolidation, and because amnesia is a cardinal endpoint of general anesthesia occurring at lower concentrations than immobility, this region is widely regarded as a primary neuroanatomical target for investigating anesthetic mechanisms (Hemmings et al., 2019; Platholi and Hemmings, 2022).

The relationship between emotional state and anesthetic sensitivity provides the rationale for the present study. We previously reported that artificial activation of the amygdala circuitry, mimicking a state of heightened anxiety or fear, significantly attenuates the inhibitory actions of general anesthetics on hippocampal synaptic transmission (Hirota et al., 2019). This finding aligns with the clinical observation that highly anxious patients often require larger doses of anesthetics (Maranets and Kain, 1999). Based on these findings, we hypothesized that EE rearing, which conversely reduces anxiety and amygdala excitability, would enhance the potency of general anesthetics in the limbic circuit. To test this hypothesis, we examined the effects of propofol and desflurane on synaptic transmission using a novel amygdala–hippocampal slice preparation that preserves the reciprocal connections between these regions. This study aims to elucidate how environmental history modulates the pharmacological profile of anesthetics at the circuit level, focusing on the GABAergic mechanisms within the amygdala–hippocampal network.

## 2 Materials and methods

### 2.1 Animals

**FIGURE 1:**
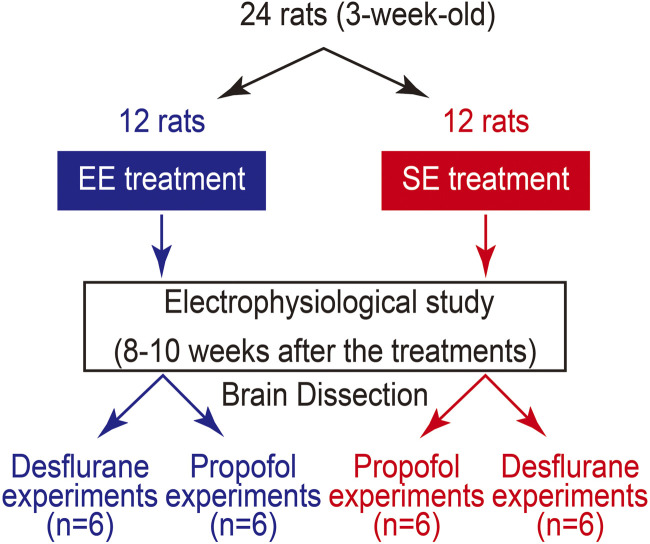
Experimental protocol. Male Wistar (3-week-old) rat pups were randomly divided into two groups, and then housed for 8–10 weeks for neuroscientific experiments.

**FIGURE 2:**
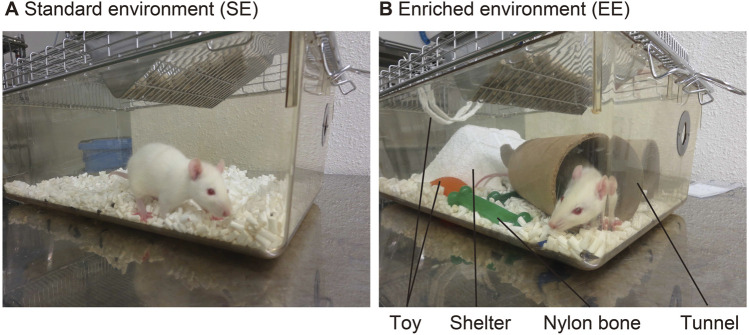
Rearing environments. **(A)** Standard environment (SE). Rats are individually housed in clear plastic cages with free access to food and water. **(B)** Enriched environment (EE): In addition to SE, various objects (toys, shelters, tunnels, nylon bones, etc.) are placed in the cage. All objects are disposable and screened for safety. Objects are kept clean, and renewed every week. The deteriorated objects are replaced as needed.

Ethical approval was obtained from the Animal Research Committee of the University of Toyama, Japan (A2019MED-31) under the direction of the ARRIVE (Animal Research: Reporting of *In Vivo* Experiments) guideline. Male Wistar rat pups (21 days old), purchased from Japan SLC, Inc. (Shizuoka, Japan), were randomly divided into two groups and housed for 8–10 weeks for neurophysiological experiments (Figure 1). To avoid social instability due to the sequential removal of cage mates, all rats were housed individually (one per cage). Rats in the standard environment (SE) group were individually housed in clear plastic cages (28 × 45 × 16 cm^3^) and raised in an air-conditioned room (20 °C) under a 12/12-h light/dark cycle, with free access to food and water (Figure 2A). For rats in the EE group, which were also housed individually, various objects (toys, shelters, tunnels, nylon bones, etc.) were placed in the cage. All objects were disposable and screened for safety. They were kept clean and renewed every week, and deteriorated objects were replaced as needed (Figure 2B).

### 2.2 Amygdala-hippocampal slice preparation

The effects of general anesthetics on the neurophysiological properties of amygdala-hippocampal slices were studied. To do this, a total of 24 amygdala-hippocampal slices were used for the electrophysiological study (Figure 1). The method for the amygdala-hippocampal slice preparation from the rats has been described previously (Hirota et al., 2019). Briefly, rats were deeply anesthetized with sevoflurane and then decapitated. Because of the lower blood/gas partition coefficient of sevoflurane (0.63), the anesthesia for decapitation should not interfere with the baseline electrophysiological recordings. The brain was removed rapidly, and 350-µm transverse slices were prepared by cutting the hemispheres vertically to the long axis 4.56–4.80 mm posterior to the bregma at an angle of 40°–45° (Paxinos and Watson, 2007) in cold, oxygenated, artificial cerebrospinal fluid (ACSF) using a Neo-Linear Slicer (DSK, Osaka, Japan). This method ensured that the amygdala-hippocampal connections were successfully maintained in the slices. The slices were then carefully removed with spatulas under a stereomicroscope and placed on a nylon mesh screen at the interface of ACSF liquid (90 mL/h) and humidified 95%O_2_/5%CO_2_ gas (1 L/min) phases in a recording chamber. To accelerate the rate of drug equilibration and to obtain stable recordings of field potentials, the liquid/gas interface brain slice chamber was modified to a minimal perfusate volume (0.8 mL). Slices were slowly warmed to 37 °C and then allowed to equilibrate for 120–180 min without electrical stimulation.

### 2.3 Electrophysiological technique and stimulus protocol

Glass microelectrodes for extracellular recordings were constructed with a P-97 micropipette puller (Sutter Instrument, Novato, CA), and the tip resistances were 3–5 MΩ when filled with ACSF. Two sets of recording microelectrodes were positioned in the region of the cell bodies and dendrites of the CA1 pyramidal neurons to record population spikes (PSs) and field excitatory postsynaptic potentials (EPSPs), respectively. A bipolar stimulating electrode (tungsten steel, coated with epoxy resin, Unique Medical, Tokyo, Japan) was placed in the region of the stratum radiatum (Rad) to stimulate the input to CA1 neurons, and a second stimulating electrode was located in the amygdala-hippocampal area (AH) to simulate the amygdala inputs to the hippocampus (Supplementary Figure S1). PS amplitudes were determined from peak positive to peak negative of the waveform. EPSP slopes were calculated by fitting digitized data points between onset and peak negativity to a linear function (dV/dt) (Supplementary Figure S2).

Square-wave stimuli (5–10 V, 50 µs), generated with a SEN-3301 stimulator (Nihon Kohden, Tokyo, Japan), were delivered to both pathways (Rad and AH) simultaneously. The minimal stimulus intensity that elicited the maximal amplitude (maximal stimulus) was used. Stimulus frequency was fixed at 0.1 Hz, since the input frequency can modify anesthetic actions (Hirota et al., 2010). Field potentials were amplified with a MEZ-8301 amplifier (Nihon Kohden) and filtered at 1 Hz–10 kHz. Analog-digital conversions of data were made at a rate of 100 kHz using a Powerlab (AD Instruments, New South Wales, Australia). The results were stored on the hard drive of a Macintosh computer (Apple, Cupertino, CA, United States), and PS amplitudes and EPSP slopes were analyzed using Scope software (AD Instruments).

As shown in Supplementary Table S1, three types of stimuli (test-pulse, pre-pulse, and tetanic-pulse) were used to elicit field potentials of CA1 pyramidal neurons and to activate amygdala circuitry. Stimuli were combined as Protocols A, B, and C to study the effects of general anesthetics on hippocampal CA1 neurons in the absence and presence of amygdala circuitry.

### 2.4 Drug application and data acquisition

From each rat, 3–4 amygdala-hippocampal slices were prepared, and one well-conditioned slice per animal was selected for the experiments. The predetermined criteria for well-conditioned slices were as follows: (a) the variability of the control PS amplitude was <5% during the initial 30-min data acquisition period, indicating that neural viability was properly maintained; and (b) the neural pathways were functionally confirmed: the Rad–CA1 connection by the elicitation of a PS > 5 mV, and the AH–CA1 connection by the presence of PS suppression upon amygdala stimulation (Supplementary Figure S1). Recovery responses were recorded for at least 30 min after washout of anesthetic-equilibrated ACSF from the chamber.

Desflurane was applied as a vapor in the pre-warmed carrier gas (95%O_2_/5%CO_2_) above the slices using calibrated commercial vaporizers (Tec 6 plus, Omeda, West Yorkshire, United kingdom). Concentrations, expressed as volume percent (vol%), refer to the dial settings on the vaporizer. Concentrations of desflurane in the perfusate of the recording chamber were determined using a portable volatile gas analyzer (OSP, Saitama, Japan): a linear relationship (1.7 × 10^−4^ M per 1.0 vol%) existed up to 15.0 vol%. Stock solutions of propofol (0.1 M) were prepared in pure dimethyl sulfoxide (DMSO) and then diluted in ACSF before perfusion into the chamber. The final concentration of DMSO (0.3%) used in the experiments did not affect the field potentials.

Since it has been reported that the time course of the onset of anesthetic action is dependent on the depth from the surface of the brain slices, and that it took 60 min to reach a steady state of action (Benkwitz et al., 2007), electrodes were placed close to the surface of the slice (<50 µm) using the hydraulically operated, three-dimensional micromanipulators (MHW-110, Narishige, Tokyo, Japan), and the anesthetics were applied 60 min before each recording.

The composition of the ACSF was (mM): NaCl 124, KCl 3.5, CaCl_2_ 2, NaH_2_PO_4_, 1.25, MgSO_4_ 2, NaHCO3 26, D-(-)-2-amino-5-phosphonopentanoic acid (AP5) 0.1, and glucose 10, prepared with purified water. The ACSF was precooled (8 °C–10 °C) and kept saturated with the 95%O_2_/5%CO_2_ gas mixture before use (pH 7.1–7.3). Since a tetanic-pulse can be anticipated to produce long-term synaptic plasticity (e.g., long-term potentiation or depression) due to N-methyl-D-aspartate (NMDA) receptor modulation, AP5 (NMDA receptor antagonist) was administered in the perfusate.

Desflurane was purchased from Baxter (Tokyo, Japan). Propofol (2,6-diisopropyl phenol) was purchased from Tanabe Pharmaceutical Co. (Osaka, Japan) and Aldrich (Tokyo, Japan). All other chemicals used were purchased from Sigma (St. Louis, MO, United States).

### 2.5 Statistical analysis

The sample size (n = 6 per group) was determined based on our previous study utilizing similar amygdala-hippocampal slice preparations (Hirota et al., 2019). In that study, the modulation of propofol (3 × 10^−5^ M)-induced inhibition by amygdala stimulation yielded a robust effect size; the population spike amplitude was 54.4% ± 12.3% under the amygdala-activated condition (Protocol II) compared to 91.2% ± 10.4% under the control condition (Protocol III), resulting in a calculated effect size (Cohen’s d) of 3.22. Based on these empirical data, we estimated a large effect size (d > 1.5) for the present study. With an alpha level of 0.05 and a power of 0.8, a sample size of 6 slices per group was calculated to be sufficient to detect significant differences. Data acquisition and analysis were not performed in a blinded manner with respect to the experimental conditions. However, to minimize bias, PS amplitude analysis was performed according to strict objective criteria (peak-to-peak determination) using digital analysis software (Supplementary Figure S2). Statistical analysis and curve fitting were performed using Prism software (GraphPad, San Diego, CA, United States). The normality of data distribution was assessed using the Shapiro–Wilk test. Comparisons of derived parameters (IC50 and Hill coefficient) between the two groups were performed using the Mann–Whitney U test, as several datasets did not follow a normal distribution. Accordingly, these quantitative data are reported as median values. Differences in concentration-response curves and recovery time courses were analyzed using two-way repeated measures analysis of variance (ANOVA), followed by Šídák’s multiple comparisons test. A P value of <0.05 was considered significant. The concentration-response curve was fitted to the Hill equation: Y = Kn/(Cn + Kn) × 100, where Y represents EPSP slope or PS amplitude (% of control), K is IC50 value, C is anesthetic concentration, and n is the Hill coefficient.

## 3 Results

**FIGURE 3:**
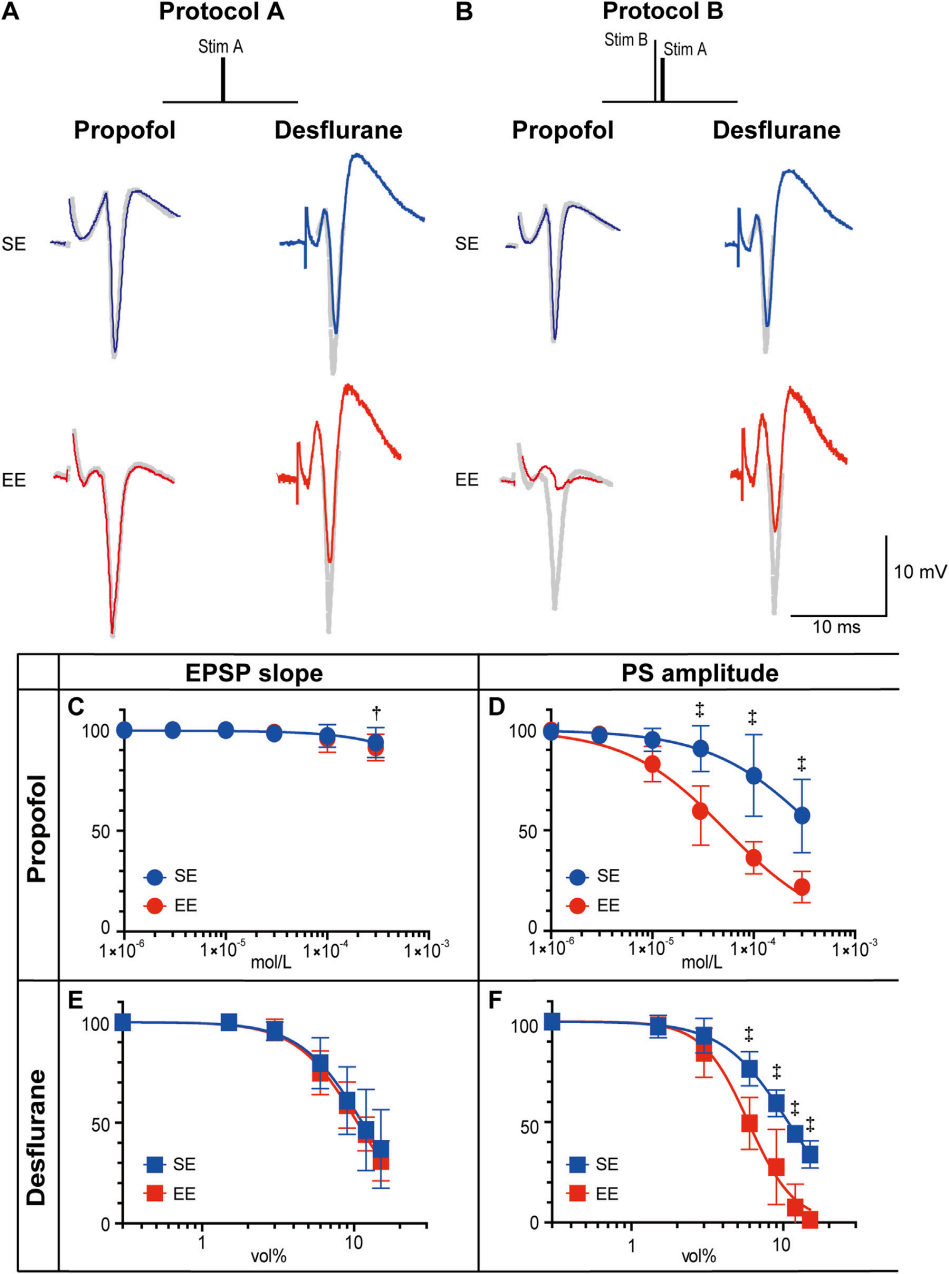
Effects of propofol and desflurane on synaptic transmission. **(A,B)** Representative recordings of population spikes (PSs) elicited with Protocol A and Protocol B in the presence of propofol (3 × 10^−4^ M) or desflurane (6 vol%). Traces from rats in a standard environment (SE) are shown in blue, and those from rats in an enriched environment (EE) are in red. Gray traces represent recordings without anesthetics (See Supplementary Figure S2 for a schematic illustration of the measurement criteria for EPSP slope and PS amplitude.). **(C–F)** Concentration–response curves for the effects of propofol on PS amplitude **(C)** and excitatory postsynaptic potential (EPSP) slope **(D)**, and for the effects of desflurane on PS amplitude **(E)** and EPSP slope **(F)**. The y-axis indicates PS amplitude or EPSP slope, expressed as a percentage of the pre-drug control value. Data are presented as mean ± SD (n = 6). The inhibitory effects on PS amplitude were significantly greater in the EE group for both propofol (P = 0.0025) and desflurane (P = 0.0021). Symbols indicate statistical significance: †P < 0.01, ‡P < 0.001 vs. SE group (two-way repeated-measures ANOVA followed by Šídák’s multiple comparisons test). To maintain clarity, symbols indicating within-group significance (i.e., concentration-dependent effects) were omitted, as the primary focus of this analysis was the inter-group comparison.

Electrophysiological studies were conducted of amygdala-hippocampal slices to determine how environmental enrichment modifies the actions of general anesthetics. Figures 3A,B shows representative recordings of PSs in the presence of either propofol or desflurane. In slices from both SE and EE rats, a standard test pulse (Protocol A) elicited a PS. When a pre-pulse was added to activate the amygdala circuitry (Protocol B), this PS was suppressed, demonstrating an inhibitory effect. The anesthetic-induced suppression of the PS was visibly more pronounced in slices from the EE group than in the SE group, suggesting enhanced sensitivity in EE rats.

To quantify these observations, concentration-response curves were generated using Protocol B. Propofol produced a concentration-dependent reduction in PS amplitude, with negligible effects on the EPSP slope (Figures 3C,D). The inhibitory action of propofol on PS amplitude was significantly greater in the EE group (main effect of group: P = 0.0025, two-way repeated measures ANOVA). The median IC50 for PS amplitude was 5.4 × 10^−5^ M [IQR: 5.1 × 10^−5^–5.6 × 10^−5^] in the EE group, which was significantly lower than 3.3 × 10^−4^ M [IQR: 2.7 × 10^−4^–3.5 × 10^−4^] in the SE group (P = 0.002, Mann–Whitney U test). The Hill coefficient did not differ significantly between groups (0.9 [IQR: 0.89–0.96] vs. 0.8 [IQR: 0.72–1.95], P = 0.39, Mann–Whitney U test). Similarly, a significant enhancement of inhibitory action was observed for desflurane in the EE group (P = 0.0021, two-way repeated measures ANOVA). Although the shift appeared less prominent than with propofol, the median IC50 was significantly lower in the EE group (6.5 vol% [IQR: 4.8–7.1]) compared to the SE group (10.4 vol% [IQR: 10.3–11.5], P = 0.002, Mann–Whitney U test). Notably, the median Hill coefficient was significantly higher in the EE group compared to the SE group (2.7 [IQR: 2.7–3.7] vs. 2.2 [IQR: 1.9–2.5], P = 0.015, Mann–Whitney U test).

**FIGURE 4:**
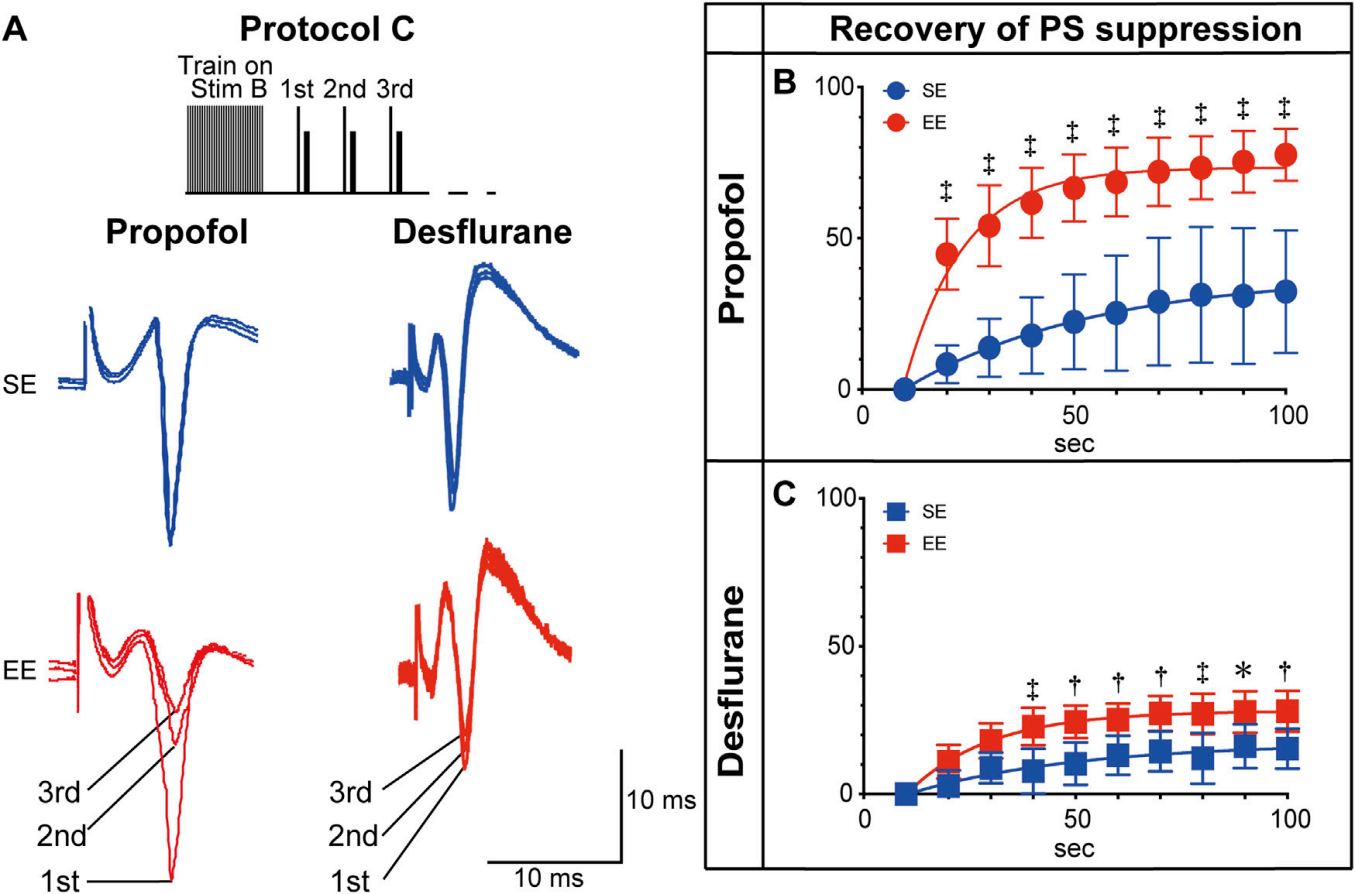
Recovery from transient disinhibition. **(A)** Representative population spike (PS) recordings elicited by Protocol C in the presence of propofol (3 × 10^−4^ M) or desflurane (6 vol%). The traces show the response to the first three stimuli following the tetanic pulse. **(B,C)** Time course of the recovery from transient disinhibition (i.e., return of PS suppression) following the tetanic pulse in Protocol C, shown in the presence of propofol **(B)** and desflurane **(C)**. Recovery was normalized such that 0% represents the maximum disinhibition (immediately after the tetanus), and 100% represents a full return to the pre-tetanus baseline suppression level (established using Protocol B). Data are presented as mean ± SD (n = 6). Recovery of PS suppression in the presence of propofol was significantly faster in the EE group than in the SE group (P = 0.0057). Symbols indicate statistical significance: *P < 0.05, †P < 0.01, ‡P < 0.001 vs. SE group at the corresponding time points (two-way repeated-measures ANOVA followed by Šídák’s multiple comparisons test). Comparison of time-dependent changes within each group was omitted to focus on the group differences in recovery kinetics.

Finally, to investigate the underlying mechanism for the enhanced anesthetic sensitivity in EE rats, Protocol C, which used a high-frequency tetanic pulse to induce a transient depletion of GABA stores (Markram and Tsodyks, 1996), resulting in disinhibition of the PS (Abbott and Regehr, 2004), was used (Figure 4A). After the tetanic pulse, the inhibitory action of the anesthetic on PS amplitude returned gradually. In the presence of propofol, this recovery was markedly faster in the EE group (main effect of group: P < 0.0057, two-way repeated-measures ANOVA), with a time constant (τ) of 36.6 s [IQR: 25.5–48.9], compared with 146.0 s [IQR: 110.6–642.8] in the SE group (P = 0.008, Mann–Whitney U test). This difference in recovery time was less pronounced in the presence of desflurane (τ = 237.8 s [IQR: 166.3–238.7] in EE vs. 450.3 s [IQR: 428.7–531.7] in SE, P = 0.056, Mann–Whitney U test).

## 4 Discussion

This study demonstrated that an EE during rearing alters the effects of general anesthetics on synaptic transmission in the amygdala-hippocampal slice, yielding two primary findings. First, an EE potentiated the effects of the anesthetics: the inhibitory effects of propofol and desflurane on PS were significantly greater in rats raised in an EE compared with those raised in a SE. Second, this potentiation was more prominent with the intravenous anesthetic propofol than with the volatile anesthetic desflurane.

The GABAergic system is central to explaining our findings, both the overall enhancement of anesthetic effects by EE and the observed differences between the two anesthetics. First, this GABA-centric mechanism accounts for the general increase in anesthetic sensitivity under an EE. Previous studies have documented the profound impact of an EE on the central nervous system, showing that an EE enhances synaptic plasticity (Eckert et al., 2010; Ohline and Abraham, 2019) and induces structural and functional changes in the GABAergic system (Nakamura et al., 1999; Segovia et al., 2006). Thus, an EE likely amplifies the effects of general anesthetics by selectively strengthening GABAergic inhibitory synapses relative to excitatory ones.

Second, this GABA-centric view is supported by the distinct pharmacological actions of the two anesthetics. In the present study, though both agents reduced PS amplitude in a concentration-dependent manner, their effects on excitatory input were distinct: propofol spared the EPSP slope (Figure 3C), whereas desflurane suppressed it (Figure 3E). These results align with prior findings that propofol primarily exerts its inhibitory neuronal effect via GABA receptors, whereas desflurane also acts on other molecular targets (Alkire et al., 2008; Asahi et al., 2006; Wakasugi et al., 1999). The Hill coefficients further illuminate the specificity of an EE’s action. For propofol, a GABA-specific agent, the coefficient remained near 1.0 in both the SE and EE groups, indicating that an EE enhanced its potency (IC50) without altering its fundamental non-cooperative mechanism. In stark contrast, for desflurane, which has multiple targets, the Hill coefficient increased significantly from 1.9 to 2.9 in the EE group, indicating a change in its cooperative binding dynamics. The fact that the mechanism of action changed only for the non-specific anesthetic (desflurane), but not for the specific one (propofol), strongly suggests that an EE’s primary effect is selective enhancement of the GABAergic system’s efficiency, while its effects on other systems are more complex and alter the very nature of drug-receptor interactions. Therefore, the stronger potentiation of propofol, a highly GABA-specific anesthetic, by an EE provides compelling evidence that an EE enhances inhibitory networks, thereby augmenting the effects of anesthetics that primarily act through the GABAergic system.

Furthermore, the present findings suggest that the inhibitory synapses of EE rats recover their function more quickly, allowing the re-establishment of the anesthetic’s suppressive effect. This was demonstrated using Protocol C, with which the recovery from transient disinhibition was markedly faster in the EE group, an effect that was particularly prominent in the presence of propofol (Figure 4B). Although the precise mechanism was not determined in this study, one possibility is an enhanced capacity for GABA synthesis. For example, previous research has shown that an EE increases the expression of the GABA-synthesizing enzyme GAD (Sale et al., 2007). Such enhancement is thought to contribute to a more robust inhibitory neurotransmitter system, a possibility that supports the present functional evidence.

The implications of the present findings may extend to clinical anesthesia. The results, demonstrating that developmental environment alters anesthetic sensitivity in rats, suggest that an individual’s life history and background could be a critical, yet currently overlooked, factor in determining anesthetic dosage in humans. For instance, a patient from a highly EE might exhibit increased sensitivity to GABAergic intravenous anesthetics like propofol. For such individuals, a standard dose could lead to an overdose, increasing the risk of adverse events like prolonged recovery or excessive hemodynamic depression. Therefore, this study highlights the potential need for a more personalized approach to anesthesia, in which patient-specific environmental factors are considered to optimize dosing and enhance perioperative safety.

A key limitation of this study is that, although we demonstrated that EE enhances anesthetic sensitivity, the precise underlying mechanism remains to be elucidated. Potential factors, such as accelerated GABA reuptake, enhanced synthesis, or increased postsynaptic receptor sensitivity, were not directly investigated, and their respective contributions will require further research.

Second, regarding the experimental subjects, although the present study was conducted exclusively in male rats, previous studies have reported significant sex differences in the anesthetic actions of propofol (Hoymork and Raeder, 2005; Choong et al., 2013) and desflurane (Tercan et al., 2005; Wasilczuk et al., 2024). While our initial focus on males was based on conventional practice, recent studies indicate that male rodents can exhibit greater intra- and inter-individual variability than females, and that diurnal fluctuations in testosterone may influence brain measures more profoundly than estrous cycle fluctuations (Prendergast et al., 2014; Kaluve et al., 2022; Levy et al., 2023). Furthermore, it has been explicitly emphasized that the effects of housing conditions should be assessed for both males and females (Simpson and Kelly, 2011). Therefore, the exclusion of females limits the generalizability of our findings across sexes, and future studies are essential to examine potential sex differences in the interaction between EE and anesthetic sensitivity.

Third, with respect to the study design, *in vivo* behavioral assays (e.g., measurement of anesthetic induction time) were not performed. In this study, we focused exclusively on the slice preparation. This approach enabled a detailed mechanistic characterization of the amygdala–hippocampal circuit, leaving *in vivo* validation as an important subject for future investigation.

Finally, concerning the scope of the enrichment model, we acknowledge that our protocol differs from standard EE paradigms, which typically involve social housing. In our experimental design, we used individual housing to standardize the environment and to avoid the potential stress associated with the sequential removal of cage mates (Burman et al., 2008). The EE paradigm in this study—consisting of toys, shelters, and tunnels—should be interpreted as a model that focuses specifically on complex sensory, cognitive, and motor stimulation, representing a distinct subset of the broader definitions of environmental enrichment (Nithianantharajah and Hannan, 2006; Sztainberg and Chen, 2010; Simpson and Kelly, 2011). It should be noted that this individual housing inherently excludes social enrichment, is known to increase anxiety (Manouze et al., 2019), and may dampen enrichment effects (Hellemans et al., 2004). Importantly, however, our results demonstrate that these physical stimuli alone were sufficiently potent to induce a robust potentiation of anesthetic effects. This finding is consistent with previous reports indicating that the benefits of environmental complexity can be retained even in the absence of social interaction (Schrijver et al., 2002).

In conclusion, EE rearing enhanced the inhibitory actions of general anesthetics on hippocampal population spikes and accelerated recovery from synaptic disinhibition, when compared with SE rearing. This modification of anesthetic sensitivity was more prominent with intravenous anesthetics than with volatile agents.

## Data Availability

The raw data supporting the conclusions of this article will be made available by the authors, without undue reservation.
