# Supplementary material for: Environmental enrichment enhances anesthetic actions in rat amygdala hippocampal circuits in vitro
Source: Front Pharmacol. 2025 Dec 19;16:1732630. doi: 10.3389/fphar.2025.1732630 (PMC12757428; doi:10.3389/fphar.2025.1732630)
Supplement: Supplementary file 1 [file Supplementaryfile1.docx]

Supplementary Material

**Figure S1**
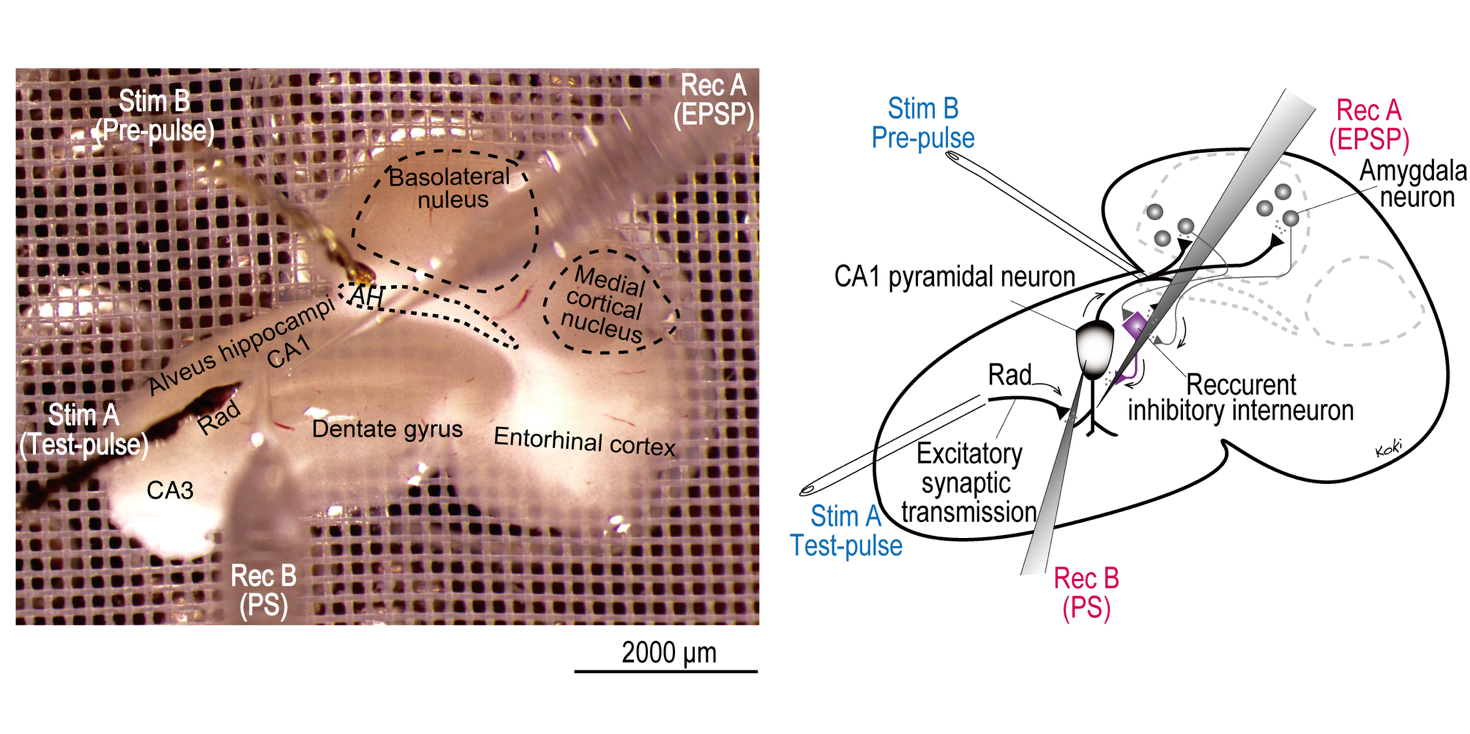


**Electrode placement in the amygdala–hippocampal slice.**
Recording electrodes were positioned in the stratum pyramidale and stratum radiatum of the hippocampal CA1 region to record population spikes (PSs) and field excitatory postsynaptic potentials (EPSPs), respectively. A stimulating electrode (A) was placed in the stratum radiatum (Rad) to activate afferent inputs to CA1 neurons, while a second stimulating electrode (B) was positioned in the amygdala–hippocampal area (AH). The inset shows a schematic diagram of the synaptic pathways in the amygdala–hippocampal slice. In the schematic on the right, the boundaries of the basolateral nucleus, medial cortical nucleus, and amygdala–hippocampal area (AH) are overlaid as light gray dotted lines to correspond with the anatomical structures in the photograph.

**Figure S2**

**
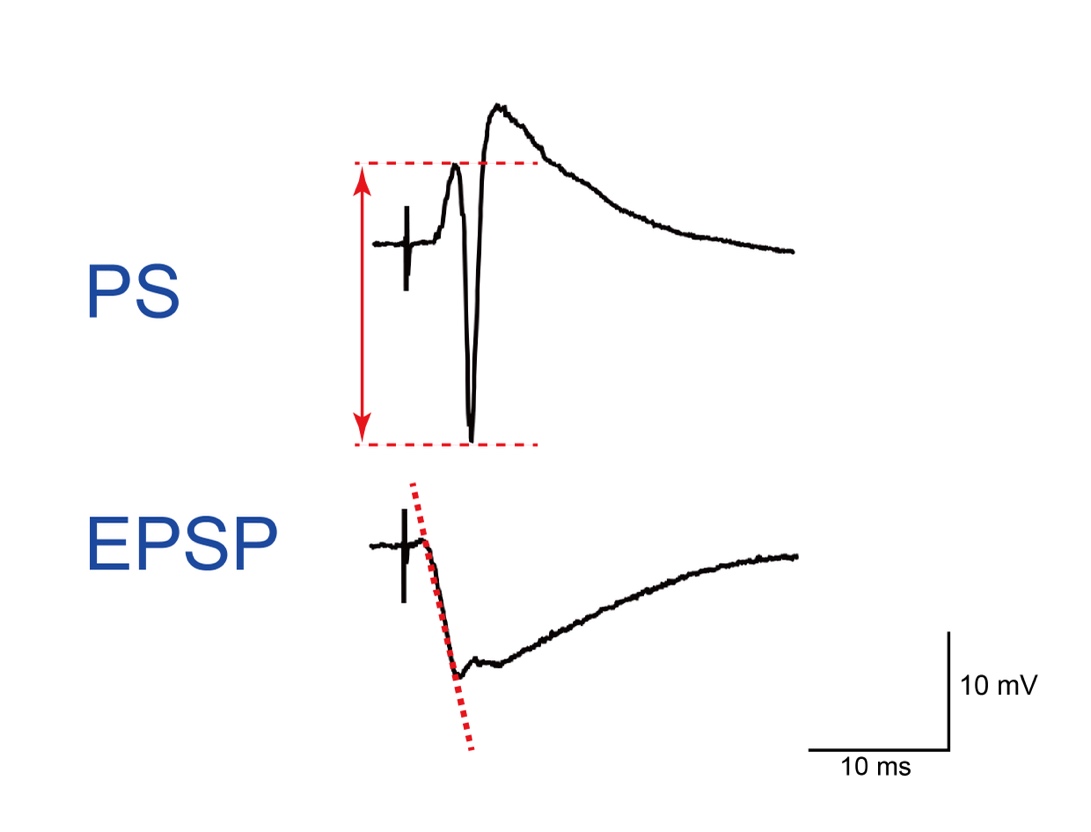
**

**Schematic illustration of waveform measurement.**A representative field potential recording showing the definitions used for data analysis. The population spike (PS) amplitude was measured as the voltage difference between the first positive peak and the subsequent negative peak (peak-to-peak amplitude). The EPSP slope was determined by linear regression of the initial rising phase of the field excitatory postsynaptic potential (between 20% and 80% of the peak amplitude), as indicated by the dashed line.

# Table S1

|  | Stimulus type | Meanings |
| --- | --- | --- |
| Test-pulse | Single 50-µs stimulus to the stratum radiatum (Rad) | Elicit hippocampal population spike (PS) and excitatory postsynaptic potential (EPSP) |
| Pre-pulse | Single 50-µs stimulus to the amygdala-hippocampal area (AH) | Ordinary activation of amygdala circuitry |
| Tetanic-pulse | High-frequency stimulation of the AH (200 Hz, 5 sec) | Maximum activation of amygdala circuitry |
|  | Stimulus sequence | Purpose |
| Protocol A | Test-pulse to Rad | Elicit hippocampal PS and EPSP in the absence of interference from amygdala circuitry |
| Protocol B | Pre-pulse to AH followed by a Test-pulse to Rad (10-ms interval) | Elicit hippocampal PS and EPSP in the presence of amygdala circuitry enhancement (single stimulus) |
| Protocol C | Tetanus-pulse to AH followed by repeated Protocol B sequences (10-s interval) | See the time-dependent changes of hippocampal PS and EPSP following maximum enhancement of amygdala circuitry |

Stimulus and experimental protocols.The table details the parameters of the stimulus types and the sequence and purpose of each experimental protocol used in the study. Abbreviations: Rad: radiatum stratum of the hippocampus, AH: amygdala-hippocampal area, PS: population spike, EPSP: excitatory postsynaptic potential.
